# Cavernous Hemangioma: A Rare Cause of Massive Lower Gastrointestinal Bleeding

**DOI:** 10.7759/cureus.10075

**Published:** 2020-08-27

**Authors:** Amna Al-Tkrit, Mohammad Aneeb, Andrew Mekaiel, Firas Alawawdeh, Asit Mehta

**Affiliations:** 1 Internal Medicine, Jamaica Hospital Medical Center, Queens, USA; 2 Gastroenterology, Jamaica Hospital Medical Center, Queens, USA

**Keywords:** hemangioma, cavernous hemangioma, small bowel hemangioma, gastrointestinal bleeding, massive lower gi bleeding, recurrent gi bleeding, benign vascular tumors, capsule endoscopy, double balloon enteroscopy

## Abstract

Due to their rarity, intestinal hemangiomas are not commonly considered as a cause of gastrointestinal (GI) bleeding. This report describes a patient who presented with massive, recurrent lower GI bleeding secondary to a cavernous hemangioma of the small intestine. The source of GI bleeding could not initially be identified despite using numerous diagnostic modalities. The lesion was eventually revealed on diagnostic laparoscopy and small bowel resection was performed.

## Introduction

Hemangiomas are benign vascular tumors that occur rarely in the gastrointestinal tract (GIT). In the GIT, the small intestine, particularly the jejunum, is the most common site of occurrence; however, only 37 cases of small intestine hemangioma were reported between the years 2000 and 2018. Small bowel hemangiomas may be a cause of massive lower gastrointestinal (GI) bleeding. Iron deficiency anemia, intestinal obstruction, abdominal pain, intussusception, or perforation are some of the other possible clinical manifestations. In this report, we present a case of massive, recurrent, acute lower GI bleeding caused by a cavernous hemangioma of the small intestine.

## Case presentation

A 32-year-old man was brought to the ED of our hospital with the primary complaints of syncope and dizziness. The patient was brought from the JFK airport by the emergency medical services (EMS) and reported that he was returning from Europe on a flight when he suddenly started feeling warm and flushed, and passed out while walking to the restroom on the plane. He stated that he was resuscitated with oxygen on the plane that made him feel better. Upon landing, the patient was evaluated by the EMS and was then brought to our hospital for further care and management.

The patient denied any similar episodes in the past but stated that he suffers from severe anxiety and occasionally becomes tachycardic. He took 10 mg of diazepam before boarding his flight, and also took two pills of peptobismol, as he felt bloated after eating. The patient denied any history of fever, chills, nausea, vomiting, chest pain, or abdominal pain. No history of NSAID use and no personal or family history of GI malignancies was present. The patient was a social drinker, an occasional smoker, and also reported occasional use of cocaine (last use was around one month prior to presentation).

On his arrival to the ED, the patient was found to have tachycardia and diaphoresis. He had a heart rate of 158 beats/min, blood pressure of 130/90 mmHg with no orthostatic hypotension, respiratory rate of 22 breaths/min, and oxygen saturation of 98%. Electrocardiogram (EKG) revealed sinus tachycardia but was otherwise normal. Laboratory investigations revealed a hemoglobin level of 12.7 g/dL (14-17 g/dL), a hematocrit of 37.3% (41%-51%), an elevated blood urea nitrogen (BUN) level of 31 mg/dL (8-20 mg/dL), and a serum creatinine of 0.8 mg/dL (0.7-1.3 mg/dL).

While in the ED, the patient had two episodes of blood-tinged dark bowel movements, after which he was transferred to the medical intensive care unit (MICU). The patient was resuscitated with IV fluids, hemoglobin and hematocrit levels were closely monitored, and a gastroenterology consultation was scheduled. The patient was started on a proton pump inhibitor infusion. Endoscopy was performed and it revealed no abnormal finding, with no source of bleeding identified. This was followed by a colonoscopy that revealed a large amount of blood with clots throughout the colon. No definite area of spurting could be identified.

**Figure 1:**
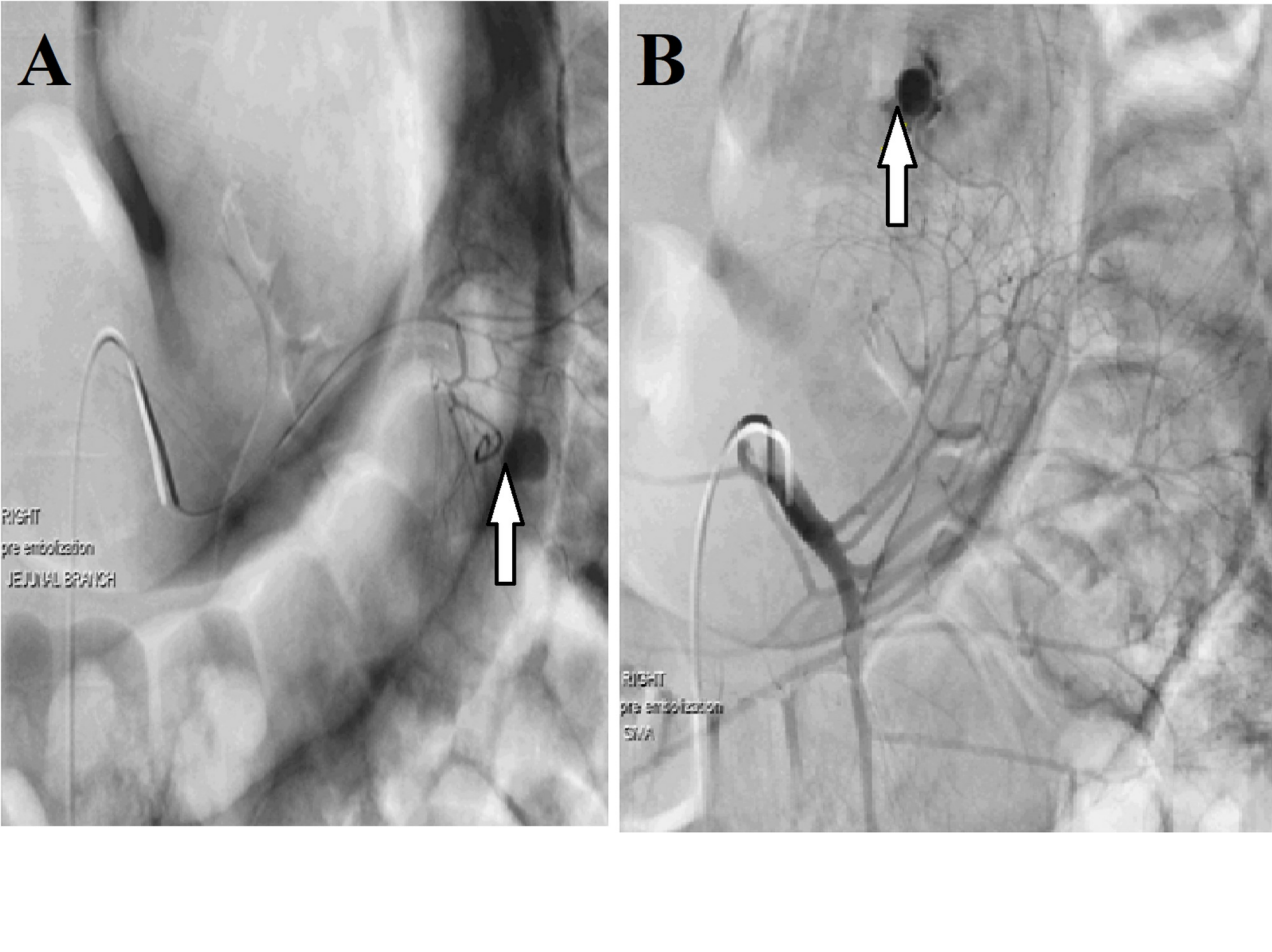
A and B: The images demonstrate brisk extravasation of contrast from a jejunal branch overlying the LUQ with flow of contrast into the small bowel. Enlarged feeding artery branches are present which suggest this is a bleeding vascular malformation. LUQ, left upper quadrant

During his stay in the MICU, the patient had frequent episodes of blood-tinged bowel movements and continued to have tachycardia. Massive transfusion protocol was activated and a total of 34 units of packed red blood cells with platelets and fresh frozen plasma were transfused. Surgery and interventional radiology were consulted, and a CT angiogram was performed that revealed no active GI bleeding. A superior mesenteric artery (SMA) arteriography was also performed, which demonstrated brisk bleeding from a jejunal branch overlying the left upper quadrant (Figure 1AB); however, the cause of bleeding remained unclear. Super selective embolization of the bleeding jejunal branch was performed and was followed by an improvement in the patient’s symptoms. The patient was later discharged from the hospital in a stable condition.

**Figure 2:**
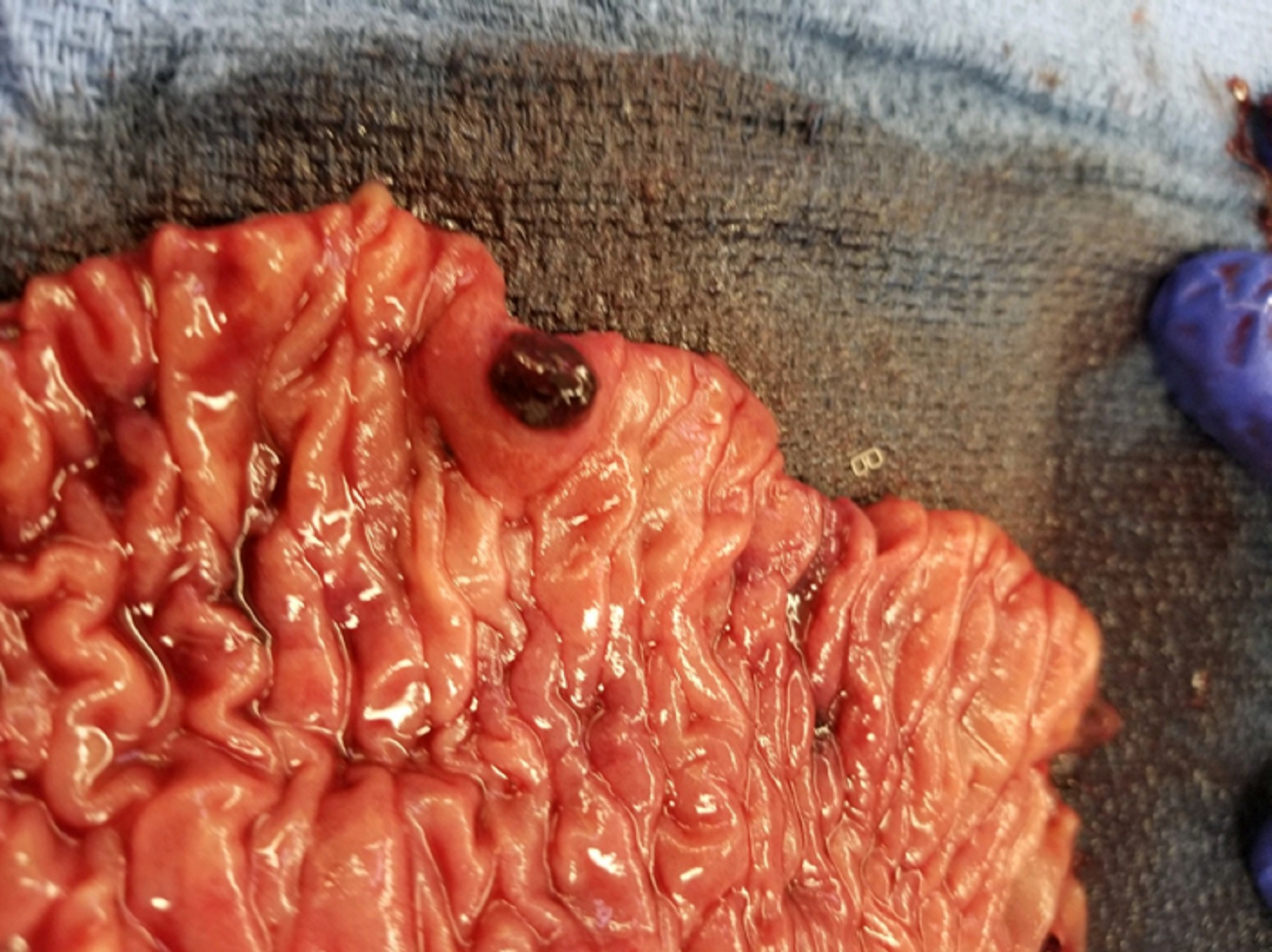
Macroscopic picture of resected small intestine showing intestinal mucosal surface with a polypoid red lesion of 1.2 cm x 1 cm x 0.8 cm.

**Figure 3:**
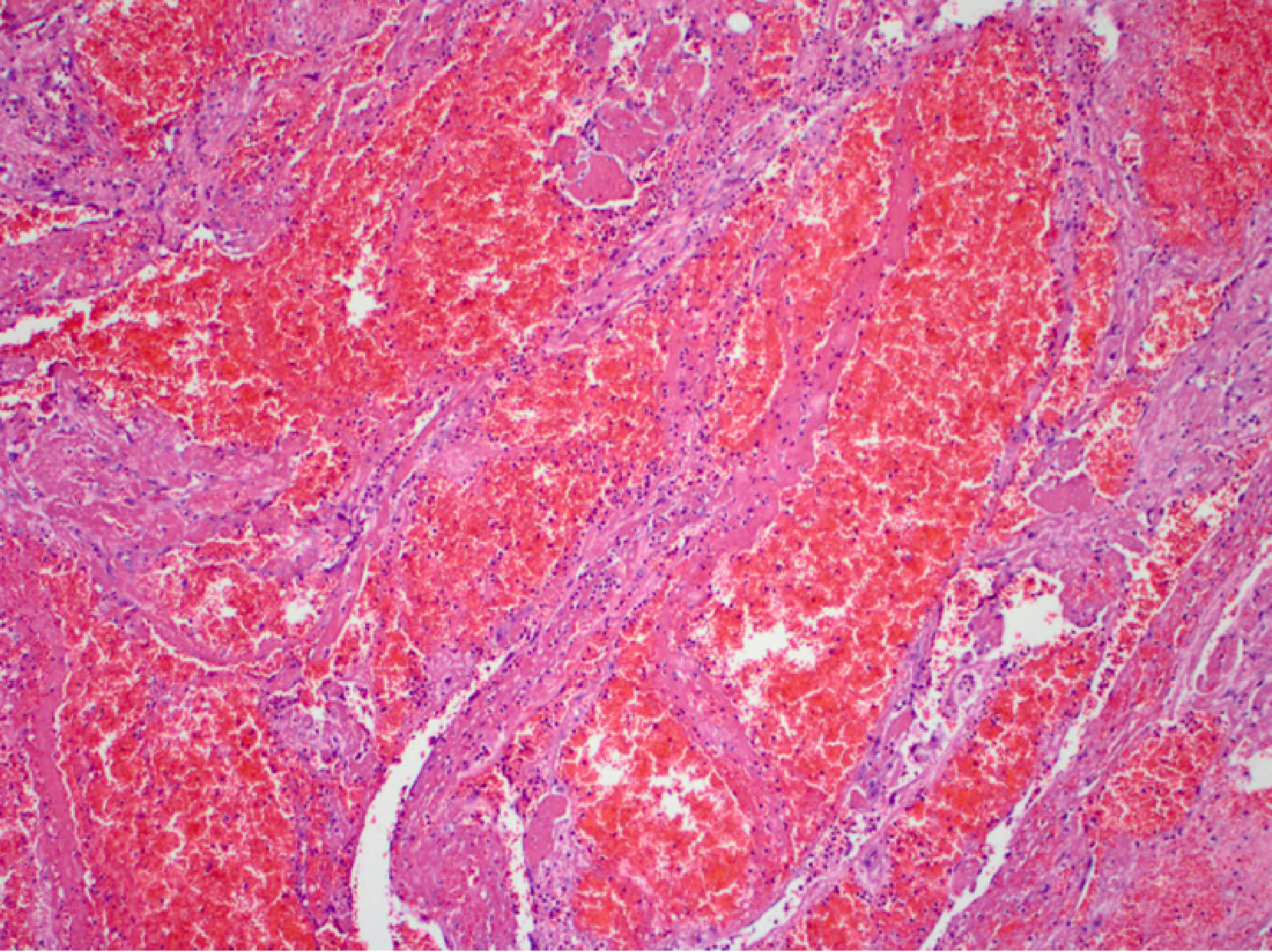
Microscopic image of the polypoid mucosal lesion exhibiting large blood-filled vascular channels with severe congestion (400x).

**Figure 4:**
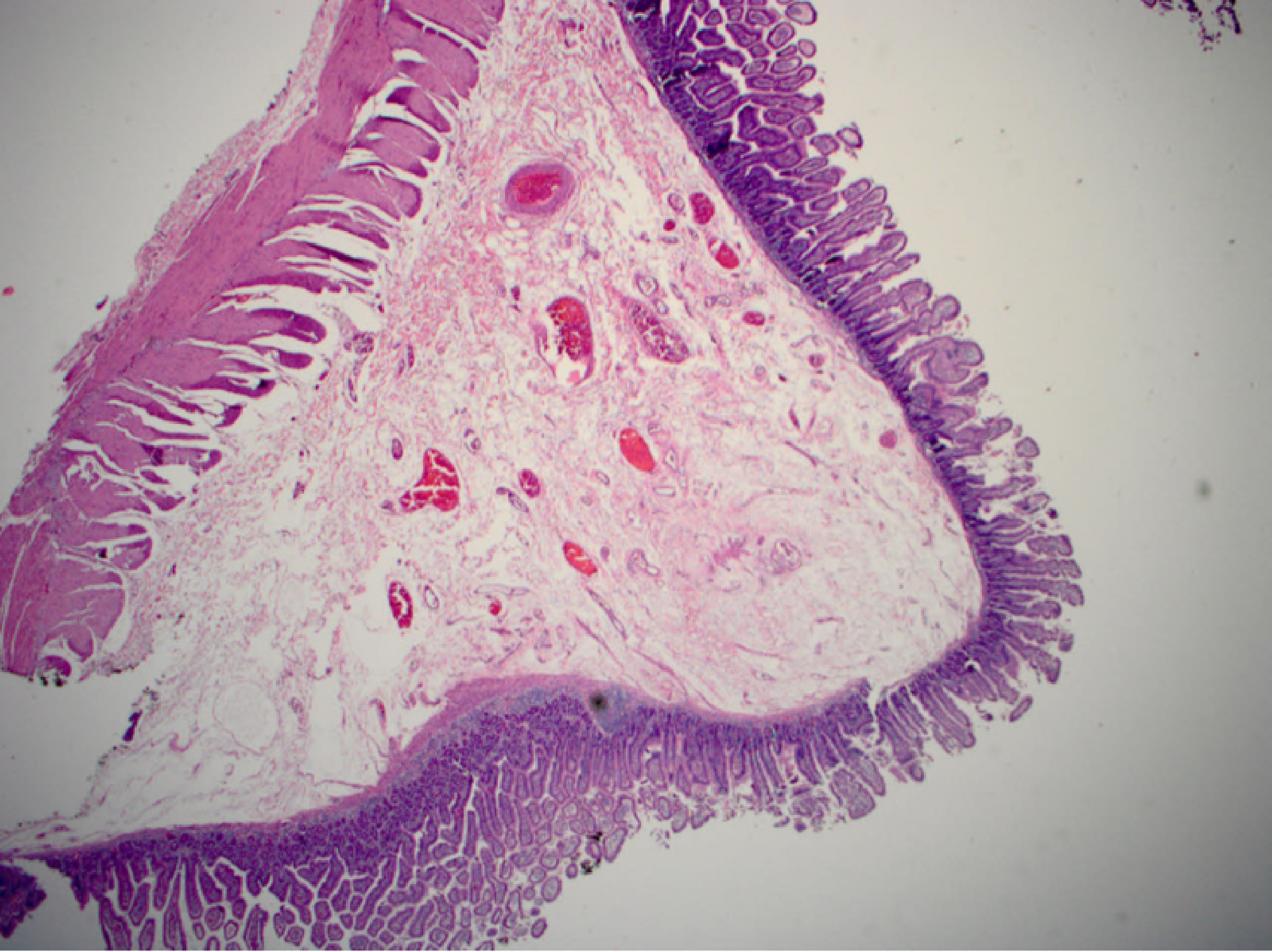
Microscopic image of normal small intestinal wall from the resected specimen (40x).

**Figure 5:**
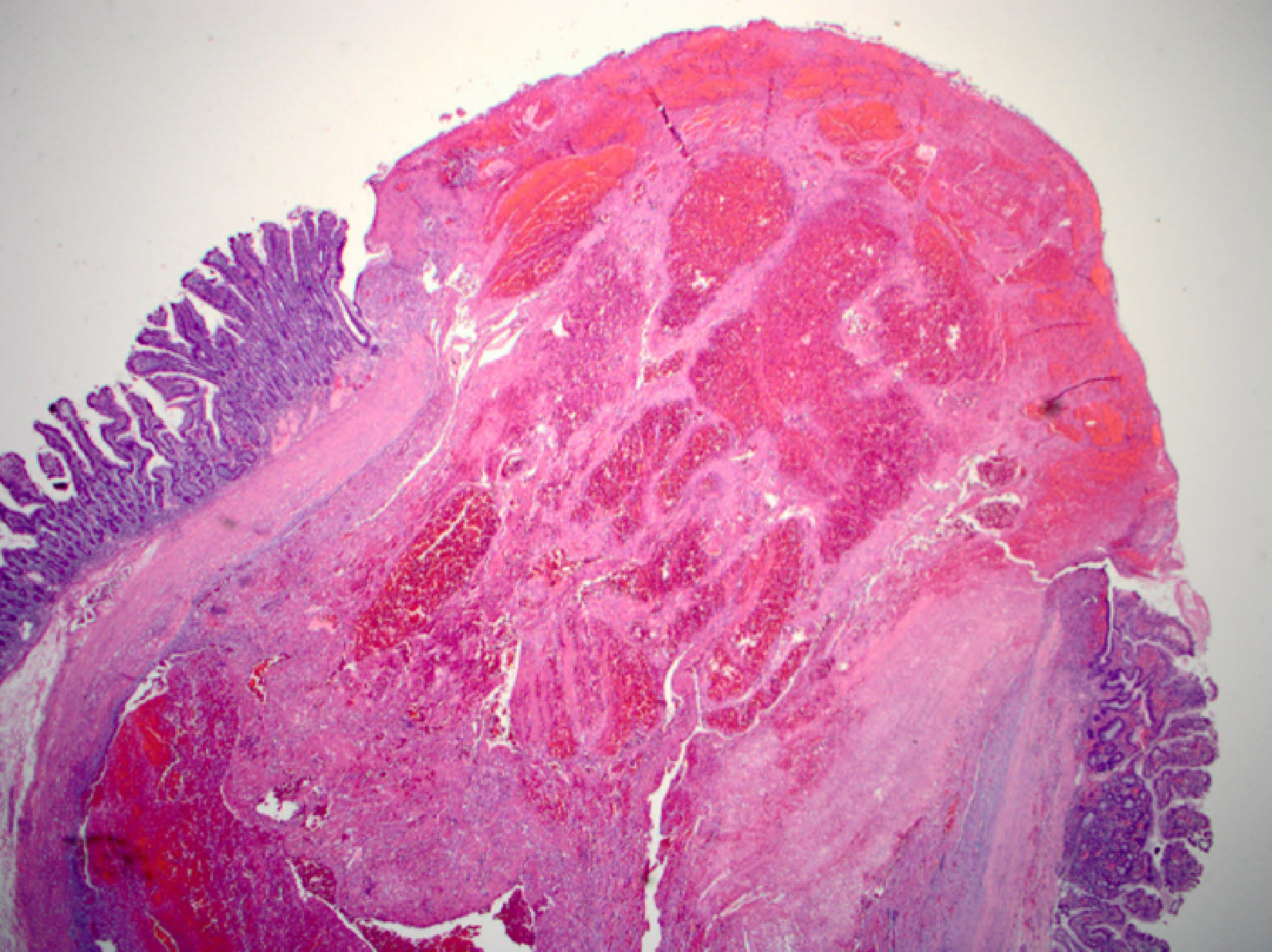
Microscopic image of the polypoid mucosal lesion exhibiting mucosal erosion and abundant blood with large vascular channels (40x).

However, after a few days, the patient was readmitted secondary to dizziness and bloody stools. Capsule endoscopy was performed and revealed active small bowel bleeding, originating most likely from the proximal- or mid-small bowel. Due to the recurrent GI bleeding that required multiple blood transfusions, the surgery team decided to perform a diagnostic laparoscopy that revealed the presence of a mass in the small intestine. Small bowel resection was performed (Figure 2), and the specimen was sent for histopathological analysis. A cavernous hemangioma, measuring 1.2 cm x 1 cm x 0.8 cm, with features of arteriovenous malformation, ulcer, and hemorrhage, was noted to be present (Figures 3-5).

## Discussion

Gastrointestinal bleeding is a potentially life-threatening condition and may result from a wide range of underlying causes. It can be classified according to the rate of blood loss, as overt (acute), occult (chronic), or obscure GI bleeding. Overt or acute GI bleeding is visible to the naked eye and may present as hematemesis, melena, or hematochezia; whereas occult or chronic GI bleeding occurs due to microscopic hemorrhage and may present insidiously as heme-positive stools. Obscure GI bleeding may either be overt or occult and is defined as recurrent bleeding with an unidentifiable source. GI bleeding may also be categorized based on the anatomical location of the source of bleeding. Bleeding originating from a site proximal to the ligament of Treitz, located at the duodenojejunal flexure, is called upper GI bleeding; and hemorrhage originating from a point distal to this ligament is referred to as lower GI bleeding [1-2].

Lower GI bleeding is common and accounts for around 20%-30% of cases of major GI bleeding. Most lower GI bleeds originate from a site distal to the ileocecal valve, and only 0.7%-9% of bleeds originate from the small intestine. Massive lower GI bleeding presents as bright red blood per rectum, known as hematochezia, and is usually seen in patients who are older than 65 years of age and have multiple medical issues. Hemodynamic instability is usually present, and the patient has a low hemoglobin level due to the acute blood loss. The mortality rate can be as high as 21% [3]. Diverticulosis is the most common cause of lower GI bleeding and accounts for over 40% cases. Hemorrhoids, ischemic colitis, inflammatory bowel disease, colon cancer, and anal fissures are some of the other causes. Vascular lesions are also a significant cause of massive, acute lower GI hemorrhage, as well as occult, chronic bleeding, particularly in older adults. Most of these lesions are arteriovenous malformations or angiodysplasia. Other vascular lesions are less commonly found and include telangiectasia, phlebectasia, and hemangiomas [4-6]. 

Hemangiomas are benign vascular neoplasms that can appear anywhere in the body, including the GIT; although their occurrence in the GIT is rare, accounting for only 0.05% of all GI tumors. In the GIT, the small intestine, particularly the jejunum, is the most common site of occurrence. Hemangiomas may be solitary or multiple. Multiple hemangiomas are usually associated with the presence of similar lesions in other organs, such as in the liver, or on the skin; and may occur as a part of certain syndromes, such as Osler-Weber-Rendu syndrome, blue rubber bleb nevus syndrome, Maffucci syndrome, and Klippel-Trenaunay-Weber syndrome [7-9].

Hemangiomas of the GIT typically originate from the submucosal vascular plexus and extend from the submucosa to the muscular layer of the intestinal wall; however, sometimes they may extend beyond the serosa, involving the mesenteric, retroperitoneal, or pelvic fat. Histologically, hemangiomas can be classified into three main types: capillary, cavernous, and mixed. Capillary hemangiomas are a proliferation of branching, thin-walled vessels of the capillary type that have a narrow lumen and are not always filled with blood. These present macroscopically as cyanotic or red nodes with a smooth or tuberous surface. Cavernous hemangiomas appear as soft, compressible, scarlet-cyanotic nodes that are separated from the surrounding tissues, and are characterized by the presence of large, irregular blood-filled sinuses or spaces (caverns), lined by either single or multiple layers of endothelial cells. A cavernous hemangioma may be a few millimeters to several centimeters in size. Mixed hemangiomas share the features of both capillary and cavernous hemangiomas. Cavernous hemangiomas are the most common of the three types and may sometimes be associated with massive, acute lower GI bleeding. Iron deficiency anemia, intestinal obstruction, abdominal pain, intussusception, or perforation are some of the other possible clinical manifestations [5, 10-11].

According to our literature review, only 37 cases of small intestine hemangiomas were reported between the years 2000 and 2018. GI bleeding and anemia were the most common manifestations seen in these cases. [12]. GI bleeding caused by small intestine hemangiomas could be massive and life-threatening; however, it may sometimes be undetectable and obscure. This can complicate the clinical picture and make the diagnostic process difficult and time-consuming.

Initial investigations in case of GI bleeding usually include upper and lower endoscopy. These techniques may be able to access some hemangiomas located in the duodenum and ileum, revealing bluish or reddish submucosal lesions that are compressible with air insufflation. However, in most cases, these investigations are normal and do not provide a clear indication of a small intestine hemangioma. Modern diagnostic modalities allow the examination of the entire intestine to help identify possible sources of bleeding and should be used in a timely manner in such cases. These imaging modalities include CT, MRI, radionuclide imaging, selective angiography, double balloon enteroscopy, and capsule endoscopy [13].

In non-contrast enhanced CT, hemangiomas appear as nodular or polypoid lesions of heterogeneous density usually with luminal growth. Phleboliths may also be seen. These are calcified nodules that develop due to thrombosis of intralesional vessels and subsequent calcification of the thrombus. If grouped together and seen in young patients, phleboliths are virtually pathognomonic of hemangiomas. Contrast-enhanced CT shows hyper vascular lesions with a peripheral and discontinuous contrast uptake in the arterial phase. Portal phase uptake is central or homogeneous. Engorgement of the mesenteric vessels located adjacent to the lesion may also be seen. MRI demonstrates wall thickening with T1-weighted images showing low signal intensity, and T2-weighted images showing high signal intensity [14]. Radionuclide imaging and selective angiography may be reserved for patients with active GI bleeding at the time of evaluation. Angiography may help confirm the diagnosis and identify pathological vessels in the mesentery [15].

Both double-balloon enteroscopy and capsule endoscopy can detect small bowel lesions at similar success rates; however, double-balloon enteroscopy is an invasive procedure that also has therapeutic potential, such as coagulation and hemostasis by clipping. Capsule endoscopy, on the other hand, is a noninvasive imaging technique that can be used routinely to identify the precise location of the lesion when the source of bleeding remains unidentified following upper and lower endoscopy [13, 16]. In certain cases, however, even the modern imaging modalities may be unable to locate the site of cavernous hemangioma. In such cases, diagnostic laparoscopy and/or laparotomy are required to confirm the presence and location of the lesion [7]. 

Surgical resection has been conventionally used for the treatment of intestinal hemangiomas. In recent years, less invasive therapeutic interventions, such as nonsurgical endoscopic treatment and minimally invasive laparoscopic surgery have become more common. Of the 37 cases of small bowel hemangiomas that were reported after the year 2000, 17 were treated endoscopically. Endoscopic mucosal resection (EMR), argon plasma coagulation, and sclerotherapy were the various endoscopic interventions used. According to the guidelines for the management of small intestine bleeding, endoscopic therapy should be used if the source of bleeding is identified. Moreover, it is more likely to be helpful in the presence of multiple, relatively small hemangiomas. However, endoscopic therapy may be associated with an increased risk of intestinal perforation, particularly, in the case of transmural intestinal hemangiomas [12, 14].

Laparoscopic surgery is another approach that can be used for the resection of a cavernous hemangioma of the small intestine. Laparoscopy can identify the affected segment of the small bowel, and if in case, open excision is needed, can allow delivery through a minimal extension of the umbilical port site. Laparoscopy can also be performed together with upper and lower GI endoscopy. This is known as laparoscopic and endoscopic cooperative surgery (LECS) and has been demonstrated as a safe technique for the resection of hemangiomas in the duodenum. The use of the laparoscopic approach results in the avoidance of an open surgery, and thus reduces the need for postoperative analgesia and allows an early return of bowel function. Open resection of the small intestine hemangioma is typically regarded as a last resort and is usually considered when the patient presents with obstructive symptoms or with life-threatening bleeding that is refractory to less invasive procedures [7, 17-18].

Despite repeated in-patient examinations with modern diagnostic procedures, the source of the recurrent lower GI bleeding in our patient remained unidentified. Due to the massive bleeding requiring multiple blood transfusions, a decision was made to perform a diagnostic laparoscopy that revealed an intestinal mass, confirmed as a cavernous hemangioma on histopathological analysis, as the source of his recurrent GI bleeding. The tumor was resected resulting in the complete resolution of symptoms.

## Conclusions

Hemangiomas are rarely found in the small intestine, and are thus, not frequently considered to be a cause of GI bleeding. Only a small number of case reports of GI bleeding caused by intestinal hemangiomas can be found in the literature. Hence, the identification of a small bowel cavernous hemangioma as a cause of massive, recurrent, acute lower GI bleeding made this a unique case. It is critical for both the endoscopists and the surgeons to consider this unusual lesion as a differential diagnosis of GI bleeding, particularly when the more common causes have been ruled out. A comprehensive workup, including modern diagnostic techniques, such as capsule endoscopy, should be performed in a timely manner to verify the diagnosis and to minimize surgical aggression by adopting a rational management approach.
